# The XPO1 Inhibitor Eltanexor Modulates the Wnt/β-Catenin Signaling Pathway to Reduce Colorectal Cancer Tumorigenesis

**DOI:** 10.1158/2767-9764.CRC-25-0052

**Published:** 2025-07-15

**Authors:** Andrew E. Evans, Sahida Afroz, Alexa Magstadt, Anup Kasi, Dan A. Dixon

**Affiliations:** 1 Department of Molecular Biosciences, University of Kansas, Lawrence, Kansas.; 2 University of Kansas Medical Center, Westwood, Kansas.; 3 University of Kansas Comprehensive Cancer Center, Kansas City, Kansas.; 4 Department of Biochemistry and Molecular Biology, Winthrop P. Rockefeller Cancer Institute, University of Arkansas for Medical Sciences, Little Rock, Arkansas.

## Abstract

Colorectal cancer is the second leading cause of cancer-related death in the United States and high-risk individuals face a notably higher likelihood of developing colorectal cancer based on their genetic background. Hence, there is a compelling need for innovative chemopreventive treatments aimed at minimizing colorectal cancer tumorigenesis. Exportin 1 (XPO1; also referred to as CRM1) plays a pivotal role in transporting proteins from the nucleus to the cytoplasm. Various cancers overexpress XPO1, including colorectal cancer, and selective inhibitors of nuclear export compounds, such as eltanexor (KPT-8602), have been developed to target XPO1. Eltanexor demonstrates fewer adverse effects than its precursors and is currently under evaluation in phase I/II clinical trials. This research evaluates eltanexor as a chemopreventive agent for colorectal cancer. Our findings indicate that eltanexor treatment inhibits expression of the common chemoprevention target in colorectal cancer, COX-2. This occurs by eltanexor-dependent reduction of Wnt/β-catenin signaling. Furthermore, XPO1 inhibition leads to forkhead transcription factor O subfamily member 3a nuclear retention, which can modulate β-catenin/TCF transcriptional activity. The *in vivo* oral treatment of eltanexor to *Apc*^*min/+*^ mice (a mouse model for familial adenomatosis polyposis) was well tolerated and reduced tumor burden by approximately threefold, along with decreased tumor size. Drug sensitivity assays using organoids from *Apc*^*min/+*^ mice tumors showed increased sensitivity to eltanexor compared with wild-type organoids. Collectively, these findings highlight XPO1 as a potent target for colorectal cancer chemoprevention.

**Significance:**

In this study, we show the XPO1 inhibitor eltanexor acts as an effective colorectal cancer chemopreventive agent both *in vivo* and *in vitro*. This occurs by reducing COX-2 expression by modulating the Wnt/β-catenin signaling pathway. Collectively, these findings highlight XPO1 as a potent target for colorectal cancer chemoprevention.

## Introduction

In the United States, colorectal cancer is the second leading cause of cancer deaths, affecting both men and women. The American Cancer Society predicts that 53,010 people will die from colorectal cancer in the year 2024 (1). These statistics highlight the clear need for novel treatment approaches to combat colorectal cancer. This need is particularly true because of the prevalence of colorectal cancer, which is expected to increase in young people. People born in the year 1990 have double the risk of developing colon cancer when compared with someone born in the year 1950 (2). In addition to younger people being more likely to develop colorectal cancer in their lifetime, they are also more likely to experience an early onset of colorectal cancer. Since 1994, the incidence of early-onset colorectal cancer (individuals younger than 50 years) has been increasing by about 2% each year (3) because of numerous risk factors.

In addition to the sporadic development of colorectal cancer, individuals with conditions such as familial adenomatous polyposis (FAP) are predisposed to colorectal cancer development because of inherited germline mutations (4). For individuals diagnosed with FAP, clinical providers recommend that they begin annual colonoscopies at the age of 10 to 12 years. For people with FAP, the risk of colorectal cancer is 100%, thus forcing many of these patients to receive a colectomy to prevent colorectal cancer (5). Given the heightened colorectal cancer risk faced by these individuals, we must develop chemopreventive agents to reduce their risk of colorectal cancer and improve their quality of life.

The nuclear export protein exportin 1 (XPO1; also known as CRM1) is crucial in transporting proteins with a leucine-rich nuclear export signal from the nucleus to the cytoplasm (6). XPO1 is overexpressed in multiple different cancer types, including colon cancer (7). The upregulation in XPO1 expression can result in the excessive removal of more than 1,000 different proteins from the cell’s nucleus. Included in those 1,000+ proteins are proteins known to be associated with the development of colorectal cancer (8, 9). To target XPO1, a novel class of drugs known as selective inhibitors of nuclear export (SINE) compounds has been developed (10). SINE compounds directly bind to the cargo-binding groove of XPO1, forming a semi-reversible covalent bond with the protein’s Cys528 (11, 12). By occupying this groove, the SINE compounds effectively inhibit XPO1’s interaction with its cargo protein (8).

A second generation of SINE compounds, eltanexor (KPT-8602), is currently in phase I/II clinical trials for multiple cancer types while demonstrating fewer side effects than precursor SINE compounds (ClinicalTrials.gov: NCT02649790; ref. 13). XPO1 inhibition impairs numerous hallmarks of cancer, including the ability to induce DNA damage and apoptosis while reducing inflammation, cell proliferation, and angiogenesis (11, 12, 14–16).

Previous research on XPO1 inhibition has demonstrated that targeting this protein reduces cell viability and proliferation, and induces apoptosis (7, 17–19). These studies have shown that XPO1 inhibition leads to the nuclear accumulation of multiple tumor suppressors and induces G_1_–S cell cycle arrest (e.g., p53, p-Rb, p21, and p27). Additionally, SINE compounds have been found to exhibit an IC_50_ in the nanomolar range and effectively limit tumor growth in colorectal cancer cell line and patient-derived xenograft models (7, 18). In a recent study, Inoue and colleagues reported that SINE compounds can induce DNA damage independently of p53 mutation status. Furthermore, their findings suggest that, in *TP53*-mutant colorectal cancer tumors, a sequential treatment regimen combining a SINE compound with an ataxia telangiectasia and Rad3-related protein inhibitor significantly improves patient-derived xenograft survival and reduces tumor burden compared with single-agent treatment (7).

Given that XPO1 is overexpressed in colorectal cancer and its inhibition reduces numerous hallmarks of cancer, we hypothesize that eltanexor will act as an effective chemopreventive agent. This report shows that eltanexor effectively limits cell viability in colorectal cancer cells, reduces the chemoprevention target COX-2, and impairs the transcriptional activity of a paramount signaling pathway in colorectal cancer, Wnt/β-catenin signaling. *In vivo*, we show that eltanexor effectively reduces tumor burden in an FAP mouse model, *Apc*^*min/+*^ mice.

## Materials and Methods

### RNA analysis

Colon cancer–staged tumor panel cDNA was purchased from OriGene (cat. #HCRT104). For normal colon tissue used in the colorectal cancer cell panel, normal tissue cDNA was obtained from OriGene and normalized to GAPDH for analysis. For all other RNA analysis, samples were normalized to actin. Total RNA from cell culture was extracted using the Qiagen RNeasy Mini Kit (cat. #74104). cDNA was synthesized using 1 μg of total RNA in combination with olgio(dT) and ImProm-II Reverse Transcriptase (Promega, cat. #A3801). qPCR using the indicated primers (Table 1) was performed as previously described (20) with SYBR green PCR master mix (Applied Biosystems, cat. #A25778) on a 7300 PCR assay system (Applied Biosystems). Fold change in mRNA expression levels was analyzed as previously described (21, 22).

### Cell culture

Colorectal cancer cells [DLD1 (RRID: CVCL_0248), HT-29 (RRID: CVCL_0320), HCT116 (RRID: CVCL_0291), MOSER (RRID: CVCL_R731), SW480 (RRID: CVCL_0546), Caco2 (RRID: CVCL_0025), and RKO (RRID: CVCL_0504)] were purchased from ATCC. HCA-7 (RRID: CVCL_0289) cells were provided by S. Kirkland (Imperial College, London, United Kingdom). DLD1, HT-29, HCT116, HCA-7, MOSER, SW480, and Caco2 were cultured in DMEM (Corning, cat. #10-013-CV), and RKO cells were cultured in minimum essential medium (Corning, cat. #10-009-CV). All media were supplemented with 10% FBS (R&D Systems, cat. #S11150) and 100 units per mL penicillin–streptomycin (Corning, cat. #30-002-CI). Cell lines were not tested for *Mycoplasma*. All cell lines were between passages 2 to 25. The cells lines DLD1, HCT116, SW480, Caco2, RKO, and MOSER are derived from males. The cell lines HCA-7 and HT-29 are derived from females. Phase-contrast images were taken using an EVOS FL Auto Imaging System. Eltanexor (APExBIO, cat. #B8335) was dissolved in DMSO (Sigma, cat. #34869) and diluted in complete media.

### Cell viability assays

Cell Counting Kit-8 (CCK8) assays were used to analyze cell viability. Into each well of a 96-well plate, 1,000 cells were plated. Twenty-four hours later, the cells were treated with varying concentrations of eltanexor (APExBIO). After 72 hours, 10 μL of CCK8 (GlpBio, cat. #GK10001) was added to each well, and the plate was incubated at 37°C for 1 hour before reading absorbance of each well at 450 nm on a BioTek ELx800 Microplate reader.

For the colony formation assay, cells were seeded at 500 cells/well in six-well plates and treated with 800 nmol/L eltanexor (APExBIO), 200 nmol/L eltanexor, or a DMSO vehicle (Sigma, cat. #472301) control. After 72 hours, the drug treatment medium was replaced with complete DMEM. DMEM was changed every 3 days for the remainder of the experiment. Eleven days after initial treatment, the cells were washed with PBS, incubated for 20 minutes in 100% methanol, and stained for 40 minutes in 0.1% crystal violet solution. Colonies were analyzed for the percentage of total plate coverage utilizing the ColonyArea ImageJ (RRID: SCR_003070) plugin https://imagej.net/plugins/colonyarea.

### Fluorescence microscopy

Cells were grown on coverslips and treated with varying dosages of eltanexor for 24 or 48 hours. Following the completion of treatment, the cells were washed once with PBS and fixed with 4% formaldehyde (Fisher, cat. #F79-500) for 15 minutes. The cells were then washed with PBS and blocked with PBS + 5% normal goat serum (Cell Signaling Technology, cat. #5425S) + 0.3% Triton X-100 (Sigma, cat. #T8787) for 1 hour. The blocking buffer was removed, and the cells were incubated with either anti-XPO1 (1/400; Cell Signaling Technology, cat. #46249S, RRID: AB_2799298), anti–forkhead transcription factor O subfamily member 3a (FoxO3a; 1/400; Cell Signaling Technology, cat. #12829S, RRID: AB_2636990), or anti–β-catenin (1/200; Cell Signaling Technology, cat. #8480S, RRID: AB_11127855) diluted in PBS + 1% BSA (Fisher, cat. #BP1600) + 0.3% Triton X-100 overnight at 4°C. The cells were then rinsed 3× with PBS. Secondary antibody was applied to the cells for 1 hour at room temperature using Alexa Fluor 488 goat anti-rabbit (1/500; Invitrogen, cat. #A11008, RRID: AB_143165) diluted in the antibody dilutant. The cells were then counterstained with DAPI (Santa Cruz Biotechnology, cat. #sc-24941) and imaged using an EVOS FL Auto Imaging System at 60× magnification.

For immunofluorescence on tissue, the tissue was formalin-fixed, paraffin-embedded (FFPE). Then 5-μm slides were cut and rehydrated by washing the samples 5 minutes/wash with xylene 2×, 100% ethanol, 95% ethanol, 80% ethanol, 50% ethanol, diH_2_O, and PBS. Antigen retrieval was performed by microwaving the slides in antigen citrate buffer pH 6.0 (Sigma, cat. #C9999) for 1 minute at high power and 5 minutes low power. The samples were then placed in a steamer for 30 minutes and then placed back in antigen citrate buffer pH 6.0 for 20 minutes. The slides were then washed for 5 minutes in PBS. Blocking buffer made of tris-buffered saline with 0.1% Tween 20 + 5% normal goat serum (Cell Signaling Technology, cat. #5425) was applied to the slides for 2 hours. The slides were then washed with PBS for 3 minutes and primary antibody diluted in blocking buffer was applied to the slides overnight at 4°C. Antibodies used included anti-XPO1 (1/200; Cell Signaling Technology, cat. #46249S, RRID: AB_2799298), anti–COX-2 (1/300; Cell Signaling Technology, cat. #12282S, RRID: AB_2571729), and anti-Ki67 (1/200; Cell Signaling Technology, cat. #12202S, RRID: AB_2620142). The slides were then washed with PBS 3×, and Alexa Fluor 488 goat anti-rabbit (1/500; Invitrogen, cat. #A11008, RRID: AB_143165) diluted in blocking buffer was applied for 2 hours. The slides were washed 3× with PBS and then incubated with Hoechst 33342 (1/1,000; Invitrogen, cat. #H3570) diluted in PBS for 5 minutes. The slides were washed 3× in PBS and incubated in 0.1% Sudan Black (Sigma, cat. #MKBB2665) diluted in 70% ethanol for 20 minutes. The slides were washed 3× with PBS and mounted using ProLong Gold Antifade (Invitrogen, cat. #P36930). The slides were imaged using an EVOS FL Auto Imaging System at 20× magnification.

### Tumor growth xenograft

HCT116 cells were thawed and passaged twice before the day of injection. On the day of injection, the cells were trypsinized, counted, and 20 million cells/ mL were resuspended in PBS. The cells were then injected into the dorsal flanks of athymic nude mice (The Jackson Laboratory). Three days after injection, the mice were randomly separated into either vehicle-treated or eltanexor-treated groups. The vehicle was comprised of 0.5% methylcellulose (Sigma, cat. #M0262) and 1% Tween 80 (Sigma, cat. #P4780). Eltanexor (APExBIO) was dissolved in the vehicle. Mice were treated with the vehicle or 10 mg/kg of eltanexor 3×/week by oral gavage. Tumor volume was monitored 3×/week by caliper. Tumor volume was calculated using the formula: tumor length × (width)^2^/2. Once the tumors reached 1,200 to 1,500 mm^3^ in size, the mice were sacrificed, the tumors were excised, and photographs were taken. The tumors were formalin-fixed and paraffin-embedded for IHC analysis.

### Western blot analysis

Western blotting was performed as described (23). The primary antibodies used included anti-XPO1 (1/1,000; Cell Signaling Technology, cat. #46249S; RRID: AB_2799298), anti-FoxO3a (1/1,000; Cell Signaling Technology, cat. #12829S, RRID: AB_2636990), anti–c-myc (1/1,000; Cell Signaling Technology, cat. #5605S, RRID: AB_1903938), anti–COX-2 (1/1,000; Cell Signaling Technology, cat. #12282S, RRID: AB_2571729), anti-actin (1/5,000; MP Biomedicals, cat. #691002, RRID: AB_2920628), and anti–β-catenin (1/1,000, Cell Signaling Technology, cat. #8480S, RRID: AB_11127855).

### DNA and siRNA transfections

Evaluation of the COX-2 promoter was accomplished using deletions of the 5ʹ regulatory region of *PTGS2* as previously described (24). TOPFlash and FOPflash luciferase reporter construct were used to evaluate Wnt/β-catenin signaling. The plasmid constructs were transfected using Lipofectamine LTX and PLUS (Invitrogen; 15338100). One day after transfection, cells were treated with varying doses of eltanexor (APExBIO) for 24 or 48 hours. The cells were then lysed and assayed as previously described (21).

For FoxO3a siRNA knockdown, FoxO3a siRNA (Horizon, cat. #L-003007-00-0005), and siRNA Control (Ambion, cat. #AM4635) were transfected into cells using siQuest (Mirus, cat. #2114). The cells were transfected with the siRNA for 24 hours, then left to recover for 24 hours, transfected again for 24 hours, and then assayed 48 hours after second transfection.

### Organoid growth and viability assays

To establish mouse organoid cultures, 15-week-old *Apc*^*min/+*^ (The Jackson Laboratory, strain #002020, RRID: ISMR_JAX002020) mice or 15-week-old wild-type (WT) C57BL/6 mice (The Jackson Laboratory, strain #000664, RRID: ISMR_JAX:000664) were euthanized. The most proximal 20 cm of the small intestines of the mice were removed, washed with PBS, and longitudinally splayed open. For the *Apc*^*min/+*^ mice, between 20 and 30 tumors were excised. For the WT mice, the small intestine was chopped into 2-cm segments using a razor in PBS. Tissue was then washed 3× with Dulbecco’s PBS (D-PBS; Corning, cat. #21-0310-CV) and 3× with D-PBS + 2% penicillin–streptomycin (Sigma) and then rocked for 90 minutes at 4°C with dissociation buffer containing 3 mmol/L EDTA (Invitrogen, cat. #AM9260G) and 0.5 mmol/L dithiothreitol (Sigma, cat. #10197777001) in D-PBS. After dissociation, the dissociation buffer was removed, and 10 mL of D-PBS was added. Tissue was pipetted 10 times; tissue was allowed to gravity settle for 30 seconds, and then the supernatant was removed and filtered through a 70-μm cell strainer (PR1MA, cat. #70ICS). This process was repeated three more times to create a total of four fractions. The fractions were then spun at 300 × *g* for 5 minutes (Eppendorf 5804 R) at 4°C. Pellets were resuspended in 10 mL of cold PBS + 0.1% (w/v) BSA (Fisher, cat. #9048-46-8). The fractions are then spun at 200 × g for 3 minutes at 4°C and pellets are resuspended in 5 mL DMEM/F12 (Cytiva, cat. #SH30023.01). For WT, the number of isolated crypts was counted in a 10 μL sample to calculate the number of crypts/mL. Furthermore, 4,000 crypts were centrifuged at 200 × *g* for 5 minutes at 4°C. Pellets were resuspended in 150 μL of Mouse IntestiCult Organoid Growth Medium (STEMCELL Technologies, cat. #06005). Then, 150 μL of Matrigel (Corning GFR, Phenol red free, LDEV Free; cat. #CB-40230C) was added to each tube, and 50 μL of crypt solution was plated into each well of a pre-warmed 24-well tissue culture non-treated plate (Falcon, cat. #351147). After 15 minutes, 750 μL of IntestiCult Media were added to each well. Media were then swapped every 2 to 3 days and organoids were stored in an incubator kept at 37°C and 5% CO_2_.

On day 5, both WT organoids and *Apc*^*min/+*^ tumor organoids were treated with varying doses of eltanexor (APExBIO) or DMSO (Sigma). The organoids were treated for a duration of 72 hours. Every 24 hours, photos of the organoids at different treatment concentrations were captured using an EVOS FL Auto Imaging System microscope at 4× magnification. Organoid size was quantified using ImageJ version 1.53k (NIH). After 72 hours, organoid media were removed and replaced with PBS containing 10 μg/mL of propidium iodide (PI; Sigma, cat. #S7109) and Hoechst 33342 (Sigma). The organoids were incubated with PBS solution at 37°C and 5% CO_2_ for 30 minutes and images of organoids on blue and red fluorescent channels were taken using an EVOS FL Auto Imaging System microscope at 4×. The number of PI-positive organoids was then scored.

### Animals

All procedures were reviewed and approved by the University of Kansas Institutional Animal Care and Use Committee. The Apc^*min/+*^ (RRID: ISMR_JAX002020) and WT mice utilized were in the C57BL/6J background (RRID: ISMR_JAX:000664) and originally purchased from The Jackson Laboratory. The colonies were maintained in a pathogen-free environment at the University of Kansas Animal Care Facility.

In the eltanexor treatment study, the vehicle used was 0.5% methylcellulose (Sigma, cat. #M0512) and 1% Tween 80 (Sigma, cat. #P1754). Seven *Apc*^*min/+*^ mice received vehicle treatment, and seven *Apc*^*min/+*^ mice received 10 mg/kg eltanexor (APExBIO) dissolved in vehicle and administered by oral gavage. The dose of 10 mg/kg was determined based on previous studies (7, 25). The sex, weight, and age of the mice prior to the start of the study were balanced. Eltanexor or vehicle administration occurred 3×/week and began when the mice were between 4 to 5 weeks old and lasted for a duration of 6 weeks. On each day, the mice received treatment and weights were recorded. Upon conclusion of the study, the mice were euthanized with isoflurane and the intestinal tract was removed, washed with PBS, longitudinally splayed, and formalin-fixed in 10% formalin (Fisher, cat. #SF100) for 24 hours and then stored in 70% ethanol. Stored tissue was given an identification code to conceal the treatment condition. Furthermore, spleens from the mice were removed and weighed.

After tissue was stored in 70% ethanol, small intestine and colon tissue were examined under a dissecting scope (Olympus SZX12). The total number of polyps and polyp sizes were determined by two individual investigators who were blinded to the treatment conditions. After counting and sizing tumors, the intestinal tissue was formalin-fixed and paraffin-embedded.

### IHC

FFPE tissue was cut into 5-µm sections. The sections were then rehydrated through a series of xylene 2×, 100% ethanol, 95% ethanol, 80% ethanol, 50% ethanol, diH_2_O, and PBS washes at 5 minutes/wash. Antigen retrieval (Sigma, cat. #C9999) was performed on the slides as described under the immunofluorescence section. The slides were then washed with PBS for 5 minutes. Then, the slides were exposed to H_2_O_2_ for 10 minutes. The slides were then washed with PBS and blocked for 2 hours using tris-buffered saline with 0.1% Tween 20 + 5% normal goat serum (Cell Signaling Technology). The slides were then exposed to anti-XPO1 (1/200; Cell Signaling Technology, cat. #46249S, RRID: AB_2799298) or anti-Ki67 (1/200; Cell Signaling Technology, cat. #12202S, RRID: AB_2620142) overnight at 4°C. The next day, the slides were washed 2× with PBS and anti-rabbit secondary antibody (SignalStain Boost IHC Detection Reagent HRP Rabbit; Cell Signaling Technology, cat. #8114, RRID: AB_10544930) was applied for 2 hours. The samples were then washed 2× with PBS for 5 minutes/wash and for 3 minutes with dH_2_O. 3,3′-diaminodbenzidine substrate solution (Cell Signaling Technology, cat. #8059S) was applied to visualize the peroxidase reaction. The slides being compared were exposed to 3,3′-diaminodbenzidine chromogen for equal durations of time. The slides were then counterstained with hematoxylin 560MX (Leica Biosystems, cat. #3801576) and mounted using Cytoseal XLT (Thermo Fisher Scientific, cat. #8312-4).

### Hematoxylin and eosin staining

FFPE tissue was sectioned into 5-µm sections. Slides were rehydrated through a series of washes in xylene and ethanol. The nuclei were stained with hematoxylin 560MX (Leica Biosystems). Bluing (Leica Biosystems, cat. #3802915) and differentiating buffers (Leica Biosystems, cat. #3803595) were applied to slides. The cytoplasm was then counterstained with eosin (Leica Biosystems, cat. #3801615). The slides were mounted using Cytoseal XLT (Thermo Fisher Scientific) and imaged with a Nikon Eclipse TE2000-U microscope.

### Statistical analysis

All graphs and statistical analyses performed in this article were done using Graphpad Prism v10.1.1 (RRID: SCR_002798). When applicable, an unpaired Student *t* test was performed, with values ≤ 0.05 considered statistically significant.

### Data availability

The data generated in this study are available upon request from the corresponding author.

## Results

### Targeting the overexpression of XPO1 in colorectal cancer

**Figure 1:**
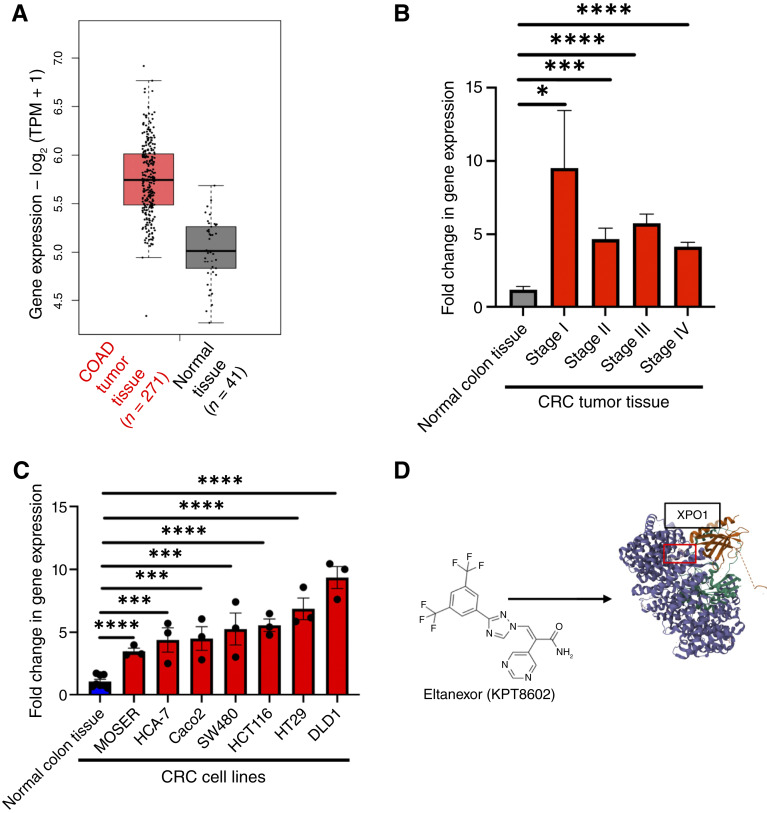
XPO1 is overexpressed in colorectal cancer. **A,** Boxplot derived from Gene Expression Profiling Interactive Analysis showing colon adenocarcinoma (COAD) tissue compared with normal colon tissue. (The Cancer Genome Atlas data derived from the online website Gene Expression Profiling Interactive Analysis: gepia.cancer-pku.cnn/detail.php?gene=XPO1). Based on a one-way ANOVA statistical test, the change in XPO1 expression is not significant. **B,** XPO1 mRNA expression by qPCR in major colorectal cancer tumor stages normalized to normal colon tissue. Actin was used as the loading control, and values represent mean fold change ± SEM. For normal human colon tissue, *n* = 8; for stage I colorectal cancer tissue, *n* = 5; for stage II colorectal cancer tissue, *n* = 9; for stage III colorectal cancer tissue, *n* = 16; and for stage IV colorectal cancer tissue, *n* = 10. A Student *t* test was used to statistically compare each colorectal cancer–staged tissue group to the normal colon tissue group. **C,** XPO1 mRNA expression by qPCR in multiple colorectal cancer cell lines compared with normal colon tissue. GAPDH was used as the loading control, and values represent mean fold change ± SEM. A Student *t* test was used to statistically compare each cell line to normal human colon tissue group. **D,** Eltanexor structure and XPO1 structure highlighting eltanexor’s protein docking region in XPO1’s protein-binding groove. (PDB: 6XJT; *, *P* ≤ 0.05; ***, *P* ≤ 0.001; ****, *P* ≤ 0.0001). CRC, colorectal cancer.

To assess XPO1’s expression in colorectal cancer tumor tissue compared with normal colon tissue, we analyzed The Cancer Genome Atlas Program data through the Gene Expression Profiling Interactive Analysis. The Gene Expression Profiling Interactive Analysis data show that XPO1 gene expression was elevated in colon adenocarcinoma tissue compared with normal colon tissue (Fig. 1A). Furthermore, we examined XPO1 expression among stage 1 to 4 colorectal cancer and found that XPO1 was consistently expressed (Fig. 1B; Supplementary Fig. S1A). The consistent overexpression of XPO1 further suggests that the overexpression is an early event in colorectal cancer. When we examined XPO1 gene expression in multiple colorectal cancer cell lines, XPO1 mRNA was consistently overexpressed in colorectal cancer cell lines examined (Fig. 1C). These data concur with previous reports showing that XPO1 is overexpressed at mRNA and protein levels in various colorectal cancer cell lines (7). When we examined the impact of XPO1 expression on overall colorectal cancer survival, we observed increases in XPO1 expression trend toward poorer survival, particularly in microsatellite-stable colorectal cancer (Supplementary Fig. S1B). These data, taken together, further suggest the possibility of XPO1 being a viable colorectal cancer chemoprevention target. The XPO1 inhibitor and second-generation SINE compound, eltanexor, inhibits XPO1 by binding to XPO1’s cargo-binding pocket, thus blocking XPO1’s ability to interact with cargo proteins (Fig. 1D). Previous studies have shown that SINE treatment leads to the nuclear accumulation of XPO1 cargo proteins (7, 26).

### Eltanexor treatment reduces colorectal cancer cell viability and *in vivo* tumorigenesis

**Figure 2:**
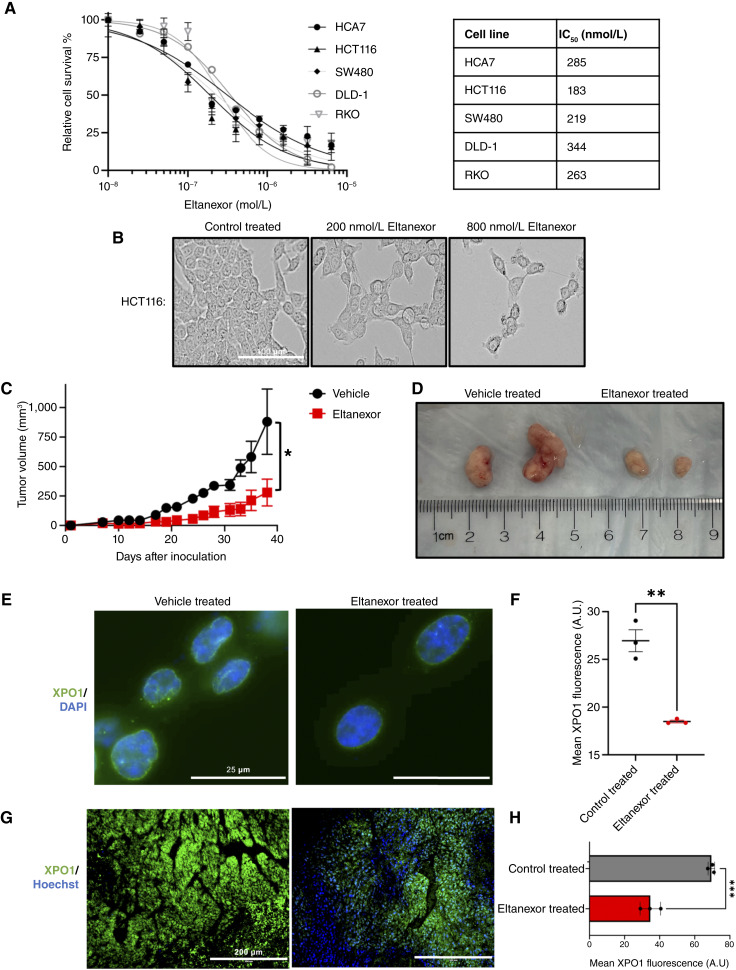
Eltanexor reduces XPO1 protein while reducing cell viability. **A,** Human colorectal cancer cell lines HCA7, HCT116, SW480, DLD-1, and RKO with varying concentrations of eltanexor. The cell survival was measured with CCK8 after incubation for 72 hours with eltanexor. The data represent the mean seven individual experiments ± SEM. The chart shows determined IC_50_ values. **B,** Phase-contrast images showing HCT116 cells treated with varying dosages of eltanexor for 48 hours. **C** and **D,** HCT116 WT cells were injected into each of the subcutaneous dorsal flanks of athymic nude mice (two tumors/mouse). Measurements were taken 3× a week. Values graphed represent mean tumor volume ± SEM. Mice were either treated with 10 mg/kg eltanexor or the vehicle 3×/week. Representative images of tumors from the vehicle-treated and eltanexor-treated mice (vehicle treated *n* = 2 mice; eltanexor treated *n* = 3 mice). A Student *t* test was performed to statistically compare the vehicle-treated tumor volume to the eltanexor-treated tumor volume. **E,** Fluorescent microscopy images of HCT116 cells treated with control or 200 nmol/L eltanexor for 48 hours. Green represents XPO1, and DAPI was used to stain the nucleus. The scale bars represent 25 μm. **F,** Comparison of XPO1 fluorescent signal between control-treated and 200 nmol/L eltanexor-treated cells. The values graphed are the mean fluorescence values of three independent experiments ± SEM. At least 40 cells were quantified for each experiment. A Student *t* test was used to statistically compare the control-treated group fluorescence to the eltanexor-treated group fluorescence. **G,** Immunofluorescent detection of XPO1 in the vehicle- and eltanexor-treated tumors. Green represents XPO1 and Hoechst was used to stain the nucleus. Representative tissue sections were used and imaged at 20× magnification. The scale bars represent 200 μm. **H**, Comparison of XPO1 fluorescent signal between vehicle-treated and eltanexor-treated tumors. The values graphed are the mean fluorescence value of three xenograft tumors ± SEM. A Student *t* test was used to statistically compare XPO1 fluorescence in vehicle-treated and eltanexor-treated tumors. A.U., arbitrary units. (*, *P* ≤ 0.05; **, *P* ≤ 0.01; ***, *P* ≤ 0.001.)

To assess the effectiveness of eltanexor in colorectal cancer, a cell viability assay was performed. Our results demonstrated that eltanexor exhibits an IC_50_ within the nanomolar range in numerous colorectal cancer cell lines with a diverse range of mutational backgrounds (Fig. 2A and B). A colony formation assay indicated that eltanexor reduces the clonogenic ability of colorectal cancer cells (Supplementary Fig. S2A and S2B). To further test whether eltanexor will impair tumorigenesis in an *in vivo* model, HCT116 cells were injected into the dorsal flanks of athymic nude mice. The eltanexor-treated mice were given 10 mg/kg eltanexor 3×/week by oral gavage, whereas vehicle-treated mice received 0.5% methylcellulose + 1% Tween 80 3×/week by oral gavage. Mice treated with eltanexor exhibited significantly reduced tumor volume compared with the vehicle-treated mice (Fig. 2C and D; Supplementary Fig. S3A). Eltanexor treatment in the xenograft mice proved to be well tolerated and reduced the common proliferation marker Ki67 (Supplementary Fig. S3B). Furthermore, XPO1 protein expression was examined in colorectal cancer cells treated with eltanexor, revealing a dose-dependent reduction with drug treatment (Fig. 2E and F; Supplementary Fig. S2C–S2F). XPO1 protein expression was also reduced *in vivo* in the drug-treated xenograft tumors (Fig. 2G and H; Supplementary Fig. S3C and S3D). Reduction of XPO1 protein following SINE treatment was consistent with previous studies in other cancer cell types which have shown that SINE compounds induce a conformational change in XPO1, leading to its recognition and degradation by the proteasome (27).

### COX-2 is reduced with eltanexor treatment

**Figure 3:**
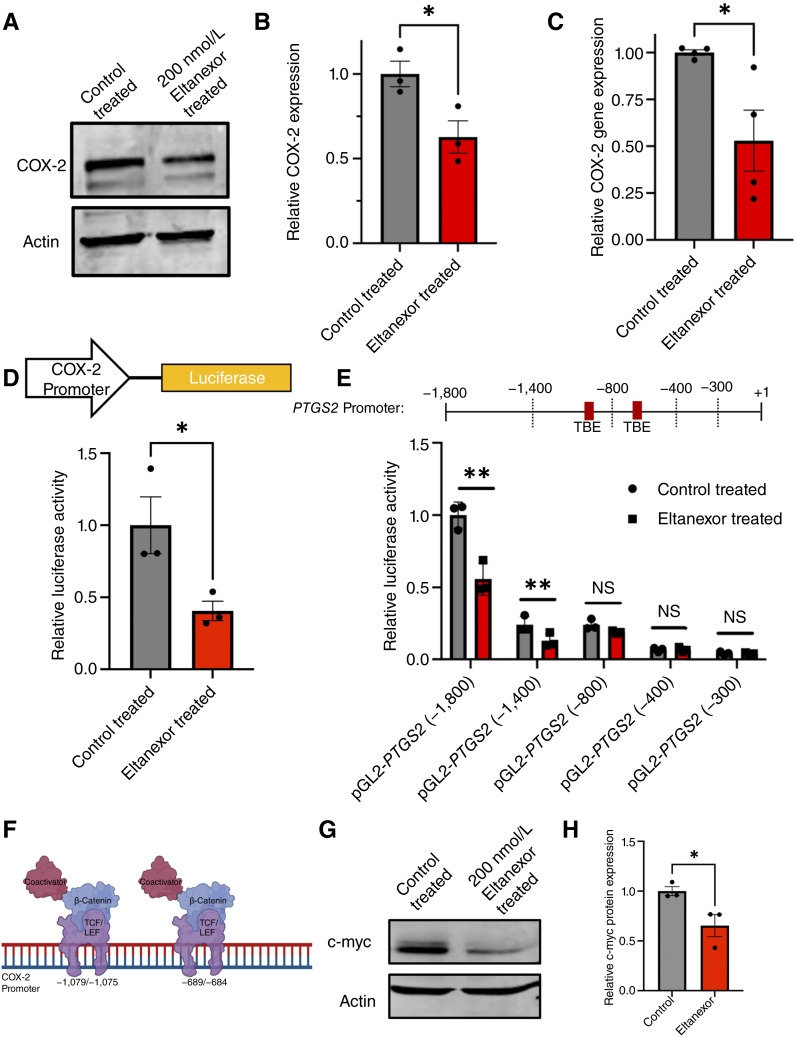
Eltanexor reduces COX-2 expression. **A** and **B,** Western blot probing for COX-2 protein in HCA-7 cells treated with either control or 200 nmol/L eltanexor for 48 hours. Actin was used as the loading control. The graph depicts the quantified densitometry of the Western blot bands. The values have been normalized to the control-treated cells. The graph shows the mean of three independent experiments ± SEM. A Student *t* test was used to statistically compare COX-2 expression in the control-treated and eltanexor-treated groups. **C,** COX-2 mRNA expression by qPCR in HCA-7 cells treated with DMSO control or 200 nmol/L eltanexor for 48 hours. The values are the mean of four experiments ± SEM. A Student *t* test was used to statistically compare COX-2 expression in the control-treated and eltanexor-treated groups. **D,** HCT116 cells transfected with a plasmid containing the COX-2 promoter + luciferase gene. The cells were subsequently treated with 200 nmol/L eltanexor for 48 hours. The graph shows the mean eltanexor-treated luciferase activity/μg of protein normalized to DMSO-treated values of three independent experiments ± SEM. A Student *t* test was used to statistically compare luciferase activity in the control-treated and eltanexor-treated groups. **E,** HCT116 cells were transfected with COX-2 promoter + luciferase gene. The promoter region was deleted from full-length down to 300 base pairs. Cells were subsequently treated with DMSO (gray bars) or 200 nmol/L eltanexor (red bars) for 24 hours. The graph shows the mean luciferase activity/μg of protein of eltanexor-treated cells normalized to DMSO-treated values of three independent experiments ± SEM. A Student *t* test was used to statistically compare luciferase activity in the control-treated and eltanexor-treated groups. **F,** Schematic representation showing the site of COX-2 expression promotion by Wnt/β-catenin signaling. **G** and **H,** Western blot probing for c-myc in HCT116 cells treated with either control or 200 nmol/L eltanexor for 48 hours. The graph depicts the mean of c-myc band densitometry value normalized to actin of three independent experiments ± SEM. A Student *t* test was used to statistically compare c-myc expression in control-treated and eltanexor-treated groups. (*, *P* ≤ 0.05; **, *P* ≤ 0.01.). NS, not significant.

To thoroughly investigate eltanexor as a chemopreventive agent, we examined whether eltanexor treatment altered COX-2 expression. COX-2 inhibition through inhibitors such as NSAIDs is frequent chemoprevention treatment options for individuals at a high risk for colorectal cancer (28). Furthermore, we have shown that COX-2 is overexpressed in colorectal cancer (29). Interestingly, when we treated HCA-7 cells with 200 nmol/L eltanexor for 48 hours, we observed a reduction in COX-2 protein and gene expression (Fig. 3A–C).

To determine whether eltanexor can induce changes at the COX-2 promoter to regulate gene expression, we utilized a luciferase reporter plasmid containing the full-length COX-2 promoter [pGL3-COX2(-1840)]. HCT116 cells were transfected with the pGL3-COX2(-1840) reporter and then treated with 200 nmol/L eltanexor for 48 hours. As shown in Fig. 3D, eltanexor reduced COX-2 promoter activity (Fig. 3D). Further analysis of promoter truncations revealed that luciferase activity in the −1,800 and −1,400 constructs was significantly reduced by eltanexor; however, the baseline activity of these constructs (untreated condition) also differed, indicating that sequence elements upstream of −1,400 may contribute to basal promoter activity independent of drug treatment (Fig. 3E). Notably, eltanexor did not significantly suppress activity from the −800 construct or shorter variants (Fig. 3E). COX-2 contains two characterized T-cell factor/lymphoid enhancer factor (TCF/LEF)–binding elements (TBE) at positions −1,079/−1,074 and −689/−684 within the promoter (Fig. 3F; refs. 30–32). Given the loss of significant repression in the shorter constructs lacking one or both TBE sites, our data suggest that these elements may contribute to eltanexor-mediated regulation. TCF/LEF transcription factors interact with a crucial protein in colorectal cancer tumorigenesis, β-catenin, to execute signaling (32). Our data suggest that eltanexor may regulate COX-2 gene expression by impairing transcriptional activity at those TBE sites. Supporting this, eltanexor also downregulates the expression of another β-catenin/TCF target, c-myc (Fig. 3G and H; ref. 33).

### Wnt/β-catenin signaling is impaired with eltanexor treatment

**Figure 4:**
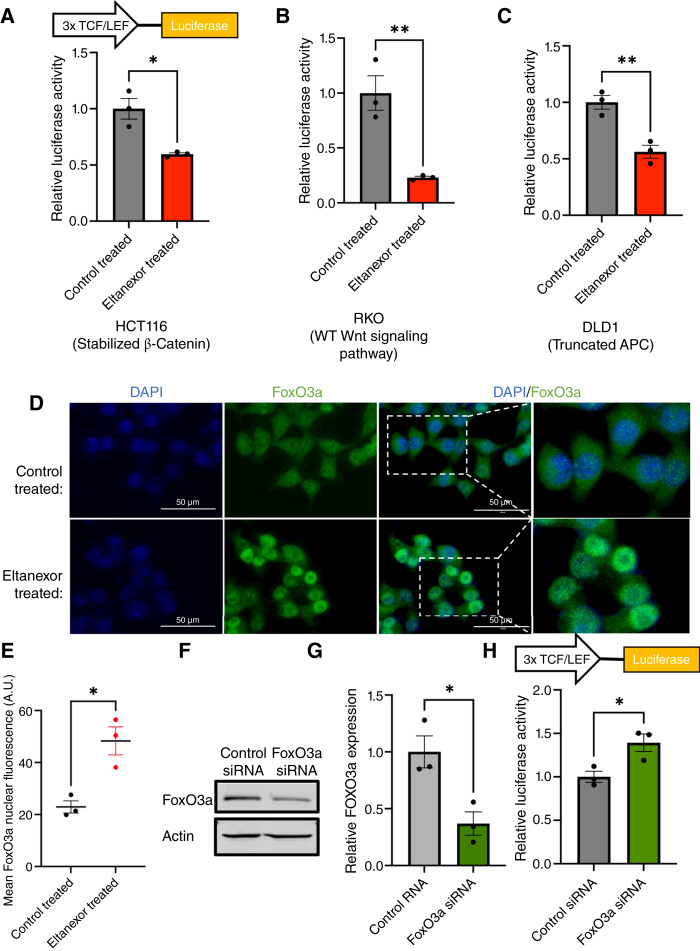
Eltanexor increases FoxO3a nuclear localization to modulate Wnt/β-catenin signaling. **A–C,** HCT116, RKO, and DLD1 cells (all harboring different Wnt signaling phenotypes) were transfected with a TOPFlash reporter plasmid. Cells were subsequently treated with DMSO or eltanexor. RKO and HCT116 cells were treated with 200 nmol/L eltanexor, and DLD1 cells were treated with 400 nmol/L eltanexor for 24 hours. Luciferase activity/μg of protein was normalized to DMSO-treated luciferase expression for each respective cell line ± SEM. A Student *t* test was used to statistically compare luciferase activity in the control-treated and eltanexor-treated groups. **D,** HCT116 cells were treated for 48 hours with either 200 nmol/L eltanexor or DMSO. Subsequently, the cells were subject to immunofluorescent staining for FoxO3a (green). The nucleus was stained with DAPI (blue). **E,** The mean FoxO3a nuclear fluorescence for cells treated with DMSO (control) or 200 nmol/L eltanexor for 48 hours. The graph depicts the mean of three independent experiments ± SEM. At least 40 cells were analyzed for each independent experiment. The scale bars represent 50 μm. A Student *t* test was used to compare the mean fluorescence in the control-treated and eltanexor-treated groups. **F** and **G,** In HCT116 cells, FoxO3a was knocked down by siRNA. After knockdown, the cells were transfected with a TOPFlash reporter plasmid. The FoxO3a knockdown–transfected and TOPFlash-transfected cells were subject to immunoblotting to confirm FoxO3a knockdown. Actin was used as the loading control. The graph depicts the quantified densitometry of the Western blot bands. The values are normalized to actin and relative to control siRNA treated. The graph depicts the mean of three independent experiments ± SEM. A Student *t* test was used to statistically compare FoxO3a expression in the control siRNA–treated and FoxO3a siRNA–treated groups. **H,** The relative luciferase activity/μg of protein values was normalized to control siRNA. A Student *t* test was used to statistically compare luciferase activity in the control-treated and eltanexor-treated groups. The graph depicts the mean of three independent experiments ± SEM. A Student *t* test was used to statistically compare the control siRNA–treated and FoxO3a siRNA–treated groups. (*, *P* ≤ 0.05; **, *P* ≤ 0.01.)

Along with being a commonly mutated pathway in sporadic colorectal cancer, the lack of regulation in the Wnt/β-catenin signaling pathway results in the development of hundreds of polyps for people with FAP (34). To determine whether eltanexor treatment can reduce Wnt/β-catenin signaling, colorectal cancer cells possessing various Wnt/β-catenin mutations were transfected with the Wnt/β-catenin signaling luciferase reporter TOPFlash. RKO possess WT Wnt/β-catenin signaling pathway and DLD1 cells have a truncated APC protein at amino acid 1,452 with WT β-catenin, whereas HCT116 cells have a β-catenin mutation due to ser45 that stabilizes the protein with WT APC (28). The colorectal cancer cells were transfected with TOPFlash and then treated with eltanexor for 24 hours. Our results revealed that regardless of Wnt/β-catenin mutational status, eltanexor can reduce Wnt/β-catenin signaling (Fig. 4A–C; Supplementary Fig. S4A–S4C). These data further suggest that XPO1 inhibition through eltanexor can regulate COX-2 expression by impairing the Wnt/β-catenin signaling pathway.

### Eltanexor increases FoxO3a nuclear location which can modulate Wnt/β-catenin signaling

To understand the reduction in Wnt/β-catenin signaling by XPO1 inhibition, we first confirmed that eltanexor does not alter β-catenin expression or intracellular localization *in vitro* (Supplementary Fig. S4D–S4G). We next searched for proteins known to interact with XPO1 and β-catenin. In doing so, we identified the tumor suppressor FoxO3a. Previous studies have shown that FoxO3a can modulate Wnt/β-catenin signaling in numerous cancer types (35–37). Furthermore, in multiple cancer types, XPO1 inhibition increases nuclear FoxO3a intracellular localization both *in vivo* and *in vitro* (27, 38). In Fig. 4D and E, we show that in colorectal cancer cells treated with 200 nmol/L eltanexor for 48 hours, FoxO3a increases in nuclear localization.

Next, we examined whether FoxO3a can modulate Wnt/β-catenin in colorectal cancer. When FoxO3a was knocked down in HCT116, there was approximately a 50% increase in Wnt/β-catenin signaling by the TOPFlash luciferase reporter (Fig. 4F; Supplementary Fig. S4H). Furthermore, when FoxO3a was knocked down, c-myc protein expression increased (Supplementary Fig. S4I and S4J). To ensure specificity of the FoxO3a siRNA, the expression of key regulators of the Wnt pathway APC, β-catenin, TCF7L1, and TCF7 was observed unchanged in FoxO3a siRNA–transfected cells (data not shown). Together, these data further highlight the ability of eltanexor to contain FoxO3a in the nucleus to modulate Wnt/β-catenin signaling.

### *Apc* ^*min/+*^ tumoroids are more sensitive to eltanexor treatment compared with WT organoids

**Figure 5:**
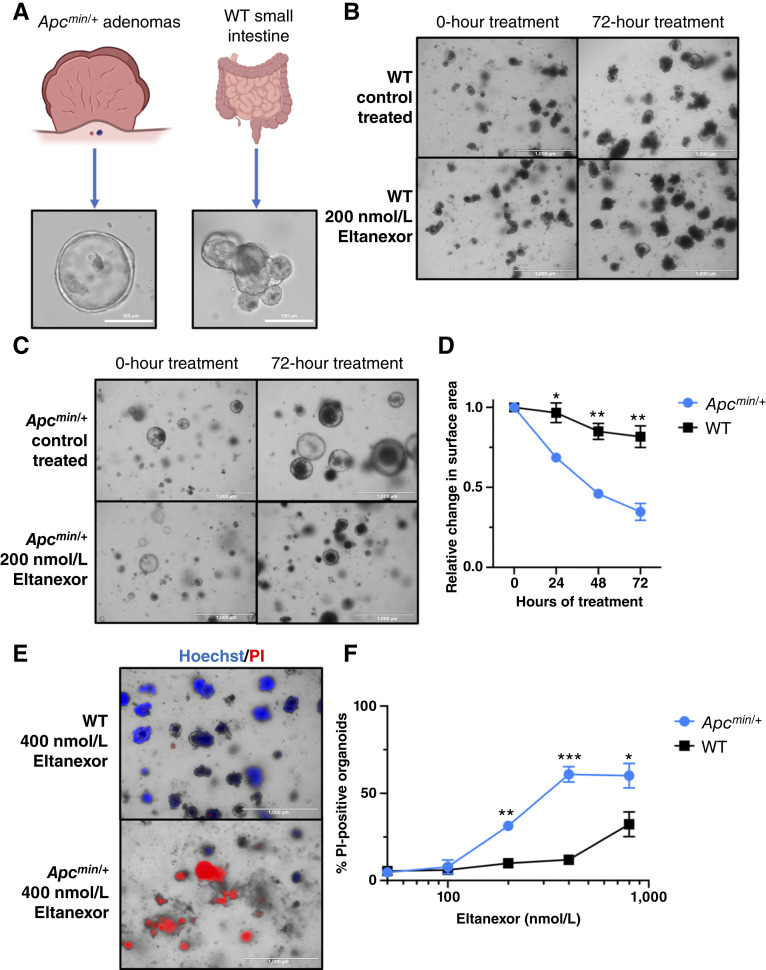
Eltanexor shows increased sensitivity to tumor-derived organoids compared with WT mouse small intestine organoids. **A,** Representative photos of *Apc*^*min/+*^ tumor–derived (tumoroids) and WT mouse small intestinal tissue–derived organoids. **B,** Representative photos of WT organoids treated with 200 nmol/L eltanexor for 72 hours. **C,** Representative photos of tumoroids treated with 200 nmol/L eltanexor for 72 hours. **D,** Tumoroids and WT organoids were treated with a dosage of 200 nmol/L eltanexor over 72 hours. Every 24 hours, the relative change in the surface area of each population was determined in ImageJ. The relative change is normalized to each population’s average surface area at hour 0. Values represent the mean of three individual experiments ± SEM. At least 50 organoids were measured at each time point under each treatment condition. A Student *t* test was used to statistically compare relative changes in surface area at each time point between control-treat and eltanexor-treated groups. **E,** Representative photos of both organoid populations that were treated with 400 nmol/L eltanexor for 72 hours. After 72 hours, each population was stained with 10 μg/mL of Hoechst and 10 μg/mL of PI. The organoids experiencing cell death will stain with PI. **F,** Tumoroids and WT organoids were treated with DMSO or 50 nmol/L, 100 nmol/L, 200 nmol/L, 400 nmol/L, and 800 nmol/L eltanexor for 72 hours. At 72 hours, the organoids were stained with 10 μg/mL of Hoechst and 10 μg/mL of PI. The graph represents the percentage of organoids from each population that was stained with PI under the various eltanexor dosages. The values represent the mean of three individual experiments ± SEM. A Student *t* test was used to statistically compare % PI-positive organoids in each treatment group between control-treat and eltanexor-treated groups. (*, *P* ≤ 0.05; **, *P* ≤ 0.01; ***, *P* ≤ 0.001.)

*Apc* ^*min/+*^ mice are a mouse model for FAP that harbors a nonsense mutation of the *APC* gene in the C57BL/6 background (39). To examine whether *Apc*^*min/+*^ mouse tumors are more sensitive to eltanexor treatment compared with WT tissue, small intestinal tumoroids from *Apc*^*min/+*^ mice and organoids from WT mice were created (Fig. 5A).

After 5 days, the tumoroids and organoids were treated with varying doses of eltanexor. Images of both organoid populations were obtained every 24 hours for 72 hours. The images were analyzed to calculate the average size of organoids from each population and dosage. Our analysis has revealed that eltanexor had a significant impact on the growth rate of tumoroids when compared with the effect it had on WT organoids (Fig. 5B–D). Furthermore, after 72 hours of treatment, the tumoroids and organoids were stained with Hoechst, which stains dead and live organoids, and PI, which only stains organoids undergoing cell death (40, 41). Then, we analyzed and scored the percentage of PI-positive organoids in each population to determine percent organoid death. Our results revealed that the tumoroids experienced a higher amount of organoid death at varying doses when compared with WT organoids (Fig. 5E and F). Altogether, these results indicate that the *Apc*^*min/+*^-derived tumoroids are more sensitive to eltanexor treatment when compared with WT organoids. These results provide evidence that FAP model mice tumors are reliant upon XPO1 for survival and growth.

### Eltanexor acts as an effective chemopreventive agent in *Apc*^*min/+*^ mice

**Figure 6:**
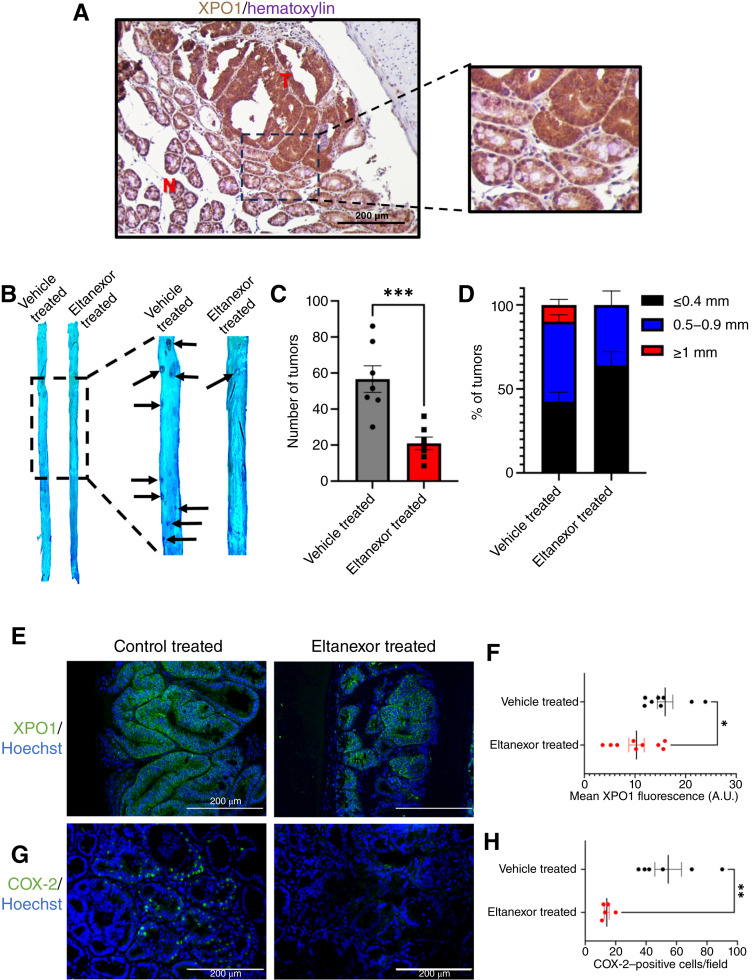
Eltanexor treatment reduces tumor burden and size in *Apc*^*min/+*^ mice. **A,** Representative photo of *Apc*^*min/+*^ mouse adenoma and adjacent normal colon IHC stained for XPO1. Nuclei are counterstained with hematoxylin. The “T” label on the tissue represents tumor tissue, and the “N” label represents normal tissue. The scale bars represent 200 μm. **B,** Seven mice received vehicle three times per week and seven mice received eltanexor three times per week at a dose of 10 mg/kg by oral gavage. Representative small intestine regions from *Apc*^*min/+*^ mice that received vehicle or eltanexor treatment. The tissue was fixed with 10% formalin and stained with 0.1% methylene blue. The arrows point to tumors present on the tissue. **C,** Total intestinal tumor burden of *Apc*^*min/+*^ mice that were treated from the age of 4 to 11 weeks with 10 mg/kg of eltanexor or vehicle 3×/week. The value represents the mean total intestinal tumor burden ± SEM of seven different mice. A Student *t* test was used to statistically compare tumor burden in vehicle-treated and eltanexor-treated mice. **D,** Frequency of ≤0.4 mm, 0.5 to 0.9 mm, and ≥1 mm tumors in the vehicle-treated and eltanexor-treated groups. **E,** Representative images and quantifications of vehicle-treated and eltanexor-treated tumors immunofluorescently stained for XPO1. Hoechst was used to stain the nucleus, and green represents the protein of interest. **F,** The quantifications are the mean fluorescent signal produced by an individual tumor. Three individual mice that received vehicle or eltanexor treatment were used in the quantification. The values represent the mean XPO1 fluorescence ± SEM. The scale bars represent 200 μm. A Student *t* test was used to statistically compare XPO1 protein expression in vehicle-treated and eltanexor-treated mice. **G,** Representative images and quantifications of vehicle-treated and eltanexor-treated tumors immunofluorescently stained for COX-2. Hoechst was used to stain the nucleus, and the green represents COX-2. **H,** The graph depicts the number of COX-2–positive cells per image. Three mice, vehicle treated or eltanexor treated, were used in the quantification. The scale bars represent 200 μm. A Student *t* test was used to statistically compare COX-2 protein expression in vehicle-treated and eltanexor-treated mice. A.U., arbitrary units. (*, *P* ≤ 0.05; **, *P* ≤ 0.01; ***, *P* ≤ 0.001.)

XPO1 is overexpressed in *Apc*^*min/+*^ tumors (Fig. 6A; Supplementary Fig. S5A). To investigate the chemopreventive potential of eltanexor, *Apc*^*min/+*^ mice were treated with 10 mg/kg of eltanexor 3×/week starting at the age of 4 to 5 weeks and for 6 weeks.

Upon completion of the study, we examined the intestinal tract of the mice for changes in tumor burden and size. Our results revealed that mice that received eltanexor drug treatment experienced an approximately threefold reduction in tumor burden (Fig. 6B and C). Effective chemopreventive compounds in *Apc*^*min/+*^ can also reduce the weight of their abnormally large spleens (42). In the eltanexor-treated mice, the weights of the spleens were significantly smaller than the vehicle-treated mice and more similar in size to WT C67BL/6 mice (Supplementary Fig. S5B). Additionally, eltanexor-treated mice have a lesser frequency of tumors greater than 0.5 mm in size (Fig. 6D). Our analysis shows that the eltanexor-treated mice maintained a comparable body weight and intestinal tract to the vehicle-treated mice (Supplementary Fig. S5C and S5D).

Furthermore, congruent with the effects we observed *in vitro*, the tumors of the mice treated with eltanexor had a reduction in XPO1 (Fig. 6E). In the eltanexor-treated tumors, we observed a reduction in the proliferation marker Ki67 (Supplementary Fig. S5E). Finally, we also observed a consistent decrease of COX-2 in the eltanexor-treated tumors (Fig. 6F). This observation further suggests the potential of eltanexor to modulate Wnt/β-catenin signaling in a mouse model to prevent colorectal cancer tumorigenesis.

## Discussion

In this study, we demonstrate the significant impact XPO1 inhibition has on colorectal cancer tumorigenesis. Our findings reveal that eltanexor treatment markedly reduces colorectal cancer cell viability by blocking XPO1’s interaction with cargo proteins and promoting subsequent XPO1 protein degradation. Furthermore, eltanexor inhibition reduces COX-2 expression, a protein previously shown to be overexpressed in both colorectal cancer adenomas and adenocarcinoma (29). COX-2 has a defined role in colorectal cancer tumorigenesis and is the predominant chemoprevention target in colorectal cancer (43–45). Our results indicate that the eltanexor-induced COX-2 reduction is mediated through modulation of the Wnt/β-catenin pathway—one of the most mutated pathways in colorectal cancer (46).

Mutations in the *APC* gene lead to aberrant Wnt/β-catenin signaling in patients with FAP, making the pathway the driver of tumorigenesis in these patients (47–49). Our results demonstrate that XPO1 inhibition not only disrupts numerous hallmarks of cancer but can also impair the signaling pathway that is the driver of tumorigenesis in FAP. Tumor-derived intestinal three-dimensional cultures from the FAP mouse model, *Apc*^*min/+*^, showed high dependency on XPO1 for survival and growth, whereas low doses of eltanexor had marginal effects on WT three-dimensional cultures. In *Apc*^*min/+*^ mice, as in patients with FAP, a germline nonsense mutation results in a truncated APC protein (39, 45, 50). Subsequently, loss of heterozygosity in these heterozygous mutants leads to the development of tumors in the small intestine and colon of the mice by approximately 4 weeks of age (51). Our preclinical evidence from *Apc*^*min/+*^ mice suggests that eltanexor may serve as an effective chemoprevention compound in patients with FAP.

Previous studies and The Cancer Genome Atlas data suggest that XPO1 is consistently overexpressed in colorectal cancer (7). Our analysis of XPO1 expression in multiple colorectal cancer stages and expression in the FAP model *Apc*^*min/+*^ mouse adenomas suggests that XPO1 overexpression is an early-occurring event in colorectal cancer tumorigenesis. XPO1 is responsible for transporting 1,000+ proteins from the nucleus to the cytoplasm (8). Because of XPO1 interacting with many proteins, the inhibition of XPO1 can lead to the impairment of multiple different hallmarks of cancer (11, 12, 14–16); we hypothesized that XPO1 is a viable colorectal cancer chemoprevention target.

To target XPO1, SINE compounds have been developed and designed to create a slowly reversible covalent bond in XPO1’s cargo-binding groove that recognizes the protein’s specific nuclear export signal (11, 12, 52). Eltanexor treatment reduced cell viability in multiple colorectal cancer cell lines with different genetic backgrounds within the nanomolar range. We additionally observed that eltanexor treatment causes a dose-dependent reduction of XPO1. Previous studies have shown that this reduction is due to proteasomal degradation of XPO1 (53). Encouragingly, these findings suggest that eltanexor can reduce XPO1 protein expression as further means to counteract the observed overexpression. We confirmed these *in vitro* findings in an *in vivo* xenograft study, which demonstrated a significant reduction in tumor volume with eltanexor treatment. Of note, tumors were not eradicated in our xenograft study. This observation is consistent with our *in vitro* data (Fig. 2B), which show that a subset of cells remains viable even after treatment with four times the IC_50_ concentration of eltanexor for 48 hours. We speculate that the residual tumor growth observed toward the end of the experiment may be due to incomplete cytotoxicity of eltanexor or the survival of resistant tumor subpopulations.

Correlations between XPO1 and COX-2 expression have been noted in ovarian cancer, in which XPO1 inhibition reduces COX-2 expression (54). Similar findings have been reported in colorectal cancer cells (55). Given these observations and the well-established role of COX-2 in colorectal cancer tumorigenesis, we sought to evaluate the effect of eltanexor on COX-2 expression. The COX enzymes contribute to the synthesis of prostaglandins. The COX enzyme isoform COX-2 is the rate-limiting step in prostaglandin E2 synthesis (45). Prostaglandin E2 can go on to perform signaling to promote tumorigenesis (56). Previous studies have found that COX-2 inhibition can reduce tumor burden in *Apc*^*min/+*^ mice and aid in reducing the risk for colorectal cancer in humans (57–60). To examine eltanexor as a chemopreventive compound, we tested whether the compound regulates COX-2 expression. Our study is the first to show that SINE compounds regulate COX-2 expression by altering transcription of COX-2. These results provide more rationale, suggesting that eltanexor will be an effective chemoprevention compound through its ability to regulate the protein that has been the chemoprevention standard for decades (44).

Although our findings suggest that COX-2 gene expression may be modulated by the Wnt/β-catenin signaling pathway, potentially through impaired β-catenin binding to TBE sites, we acknowledge that our current data do not definitively demonstrate this mechanism. To address this, we are developing luciferase reporter constructs containing the COX-2 promoter with site-directed mutations at the TBE sites. These constructs will allow us to rigorously assess whether TBE elements are required for eltanexor-dependent suppression of COX-2 promoter activity in future studies. Based on our preliminary data, we hypothesize that mutating these TBE sites will diminish or abolish the response to eltanexor.

Eltanexor can reduce the transcriptional activity of the Wnt/β-catenin signaling pathway, supporting its potential as a chemopreventive agent in colorectal cancer. We show that total β-catenin levels are not altered following eltanexor treatment. Furthermore, immunofluorescence staining indicates no observable change in intracellular β-catenin localization. However, to conclusively determine eltanexor’s effect on β-catenin intracellular distribution, future studies involving immunoblotting of nuclear and cytoplasmic fractions will be conducted. FoxO3a, which modulates the canonical Wnt/β-catenin signaling pathway by competitively binding to β-catenin in the nucleus (35–37), exhibits increased nuclear localization in eltanexor-treated cells. This suggests that the changes in FoxO3a subcellular localization contribute to the reduction of Wnt/ β-catenin signaling.

*In vivo*, COX-2 expression can be induced by numerous factors, including numerous immunogenic and oncogenic factors (45). Notably, in the *Apc*^*min/+*^ mouse model, we primarily observed changes in stromal COX-2 protein expression. Although our *in vitro* data suggest that Wnt/β-catenin signaling is involved in controlling COX-2 gene expression, further *in vivo* studies evaluating this are ongoing to determine the extent of COX-2 reduction in *Apc*^*min/+*^ mice by eltanexor to determine whether it is occurring through inhibition of the Wnt/β-catenin pathway.

Notably, *Apc*^*min/+*^ tumoroids exhibit increased sensitivity to eltanexor treatment compared with WT organoids. The heightened sensitivity to eltanexor suggests that eltanexor may be well tolerated and effective at limiting colorectal cancer tumorigenesis in an *in vivo* model. When we tested eltanexor in the *Apc*^*min/+*^ mice, we were encouraged to see similar results as our *ex vivo* study. The *Apc*^*min/+*^ mice given eltanexor experienced reduced tumor burden and size while well tolerating the drug. These results show that *Apc*^*min/+*^ tumors rely on XPO1 for viability and growth.

One possible explanation for this differential sensitivity is that *Apc*^*min/+*^ tumor cells, due to their constitutive activation of the Wnt/β-catenin pathway, may be more dependent on this signaling axis for proliferation and survival than WT epithelial cells. As eltanexor disrupts nuclear export and modulates key transcriptional programs, including Wnt target gene expression, this dependence may render *Apc*^*min/+*^ tumors particularly vulnerable to XPO1 inhibition. This mechanistic vulnerability is a promising avenue for therapeutic exploitation. To a similar effect, previous studies have shown β-catenin to be effect at limiting *Apc*^*min/+*^ burden while being well tolerated (61).

Furthermore, a recent study showed that an XPO1-derived peptide acts as an activator for NK cells in hepatocellular carcinoma (62). This study and others highlight the impact XPO1 overexpression and inhibition has on the tumor microenvironment. Because of our findings that tumoroids are more sensitive to eltanexor treatment compared with WT organoids, we chose to focus on eltanexor’s effects on the epithelial tumor tissue. However, future studies will aim to further characterize the alteration in the tumor microenvironment by eltanexor in hopes to elucidate any changes in immunogenic factors that may be aiding the compound’s ability to half colorectal cancer tumorigenesis.

Given XPO1’s involvement in multiple pathways related to colorectal cancer progression, and our *in vivo* and *in vitro* evidence demonstrating that eltanexor inhibits a broad range of colorectal cancer cells, we speculate that eltanexor could serve as an effective chemopreventive agent for other individuals who are at a high risk for colorectal cancer. Moreover, individuals with inflammatory bowel disease and Lynch syndrome have experienced colorectal cancer chemoprevention benefits from COX-2 inhibitors (63–66). Future preclinical studies are necessary to evaluate eltanexor’s effectiveness in these patient populations.

This study provides compelling preclinical evidence for eltanexor’s potential as a chemopreventive compound for patients with FAP. Future studies should focus on further evaluating the safety and efficacy of eltanexor as a chemopreventive agent in this patient population. These studies will include single-cell RNA sequencing to thoroughly assess the key pathways altered in both tumor and normal tissues. Given XPO1’s involvement in numerous pathways related to colorectal cancer tumorigenesis, eltanexor holds promise as a broad-spectrum chemopreventive agent for high-risk patient populations.

**Table 1:** qPCR primer sequences

Gene	Forward (5ʹ–3ʹ)	Reverse (5ʹ–3ʹ)
*ACTIN*	TGG​TTA​CAG​GAA​GTC​CCT​TGC​C	ATG​CTA​TCA​CCT​CCC​CTG​TGT​G
*XPO1*	AAG​GAG​CCC​AGC​AAA​GAA​T	CAT​TTT​CCA​AAA​TTT​GTA​GTC​CA
*GAPDH*	CCA​CTC​CTC​CAC​CTT​TGA​CG	CCA​CCA​CCC​TGT​TGC​TCT​AG
*COX-2*	GCA​CTA​CAT​ACT​TAC​CCA​CTT​CA	GCC​ATA​GTC​AGC​ATT​GTA​AGT​TG

## Supplementary Material

Supplementary Figure 1 Supplementary Figure 1. XPO1 expression is similar amongst all stages of CRC and overexpression trends towards worst prognosis in MSS CRC.

Supplementary Figure 2 Figure S2. Eltanexor treatment reduces XPO1 protein expression in multiple CRC cell lines and prevents colony formation.

Supplementary Figure 3 Figure S3. Eltanexor treatment reduces HCT116 xenograft Growth.

Supplementary Figure 4 Figure S4. Eltanexor does not changes β-catenin protein expression or intracellular localization.

Supplementary Figure 5 Figure S5. Eltanexor-treatment is well-tolerated in Apcmin/+ mice.
