# Supplementary material for: The XPO1 Inhibitor Eltanexor Modulates the Wnt/β-Catenin Signaling Pathway to Reduce Colorectal Cancer Tumorigenesis
Source: Cancer Res Commun. 2025 Jul 15;5(7):1140–54. doi: 10.1158/2767-9764.CRC-25-0052 (PMC12260813; doi:10.1158/2767-9764.CRC-25-0052)
Supplement: Supplementary Figure 1 — XPO1 expression is similar amongst all stages of CRC and overexpression trends towards worst prognosis in MSS CRC. [file crc-25-0052_supplementary_figure_1_suppsf1.pdf]

A

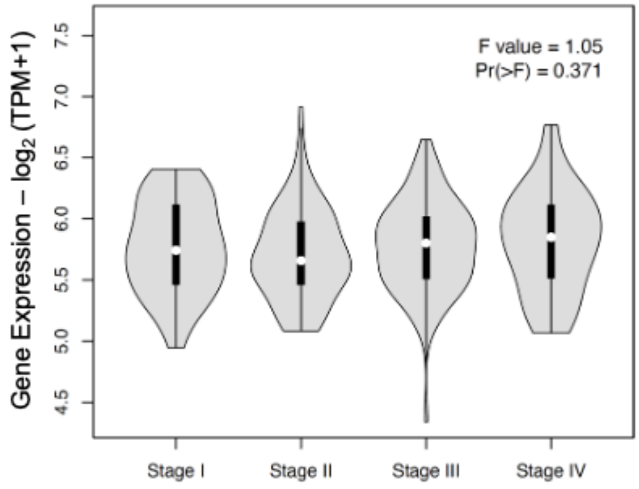

B

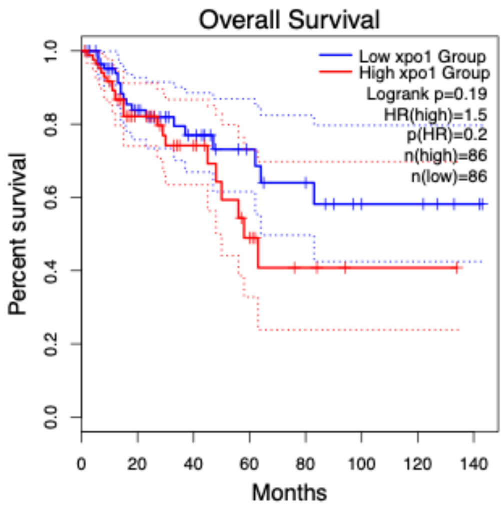

Supplementary Figure 1. XPO1 expression is similar amongst all stages of CRC and overexpression trends towards worst prognosis in MSS CRC (A) Analysis of XPO1 expression amongst CRC tumor stages derived from GEPIA (http://gepia2.cancer-pku.cn/#analysis). **(B)** Kaplan-Meier plot derived from GEPIA showing colon adenocarcinoma (COAD) microsatellite stable patient survival with high or low XPO1 expression (GEPIA: http://gepia2.cancer-pku.cn/#survival)
