# Supplementary material for: The XPO1 Inhibitor Eltanexor Modulates the Wnt/β-Catenin Signaling Pathway to Reduce Colorectal Cancer Tumorigenesis
Source: Cancer Res Commun. 2025 Jul 15;5(7):1140–54. doi: 10.1158/2767-9764.CRC-25-0052 (PMC12260813; doi:10.1158/2767-9764.CRC-25-0052)
Supplement: Supplementary Figure 2 — Figure S2. Eltanexor treatment reduces XPO1 protein expression in multiple CRC cell lines and prevents colony formation. [file crc-25-0052_supplementary_figure_2_suppsf2.pdf]

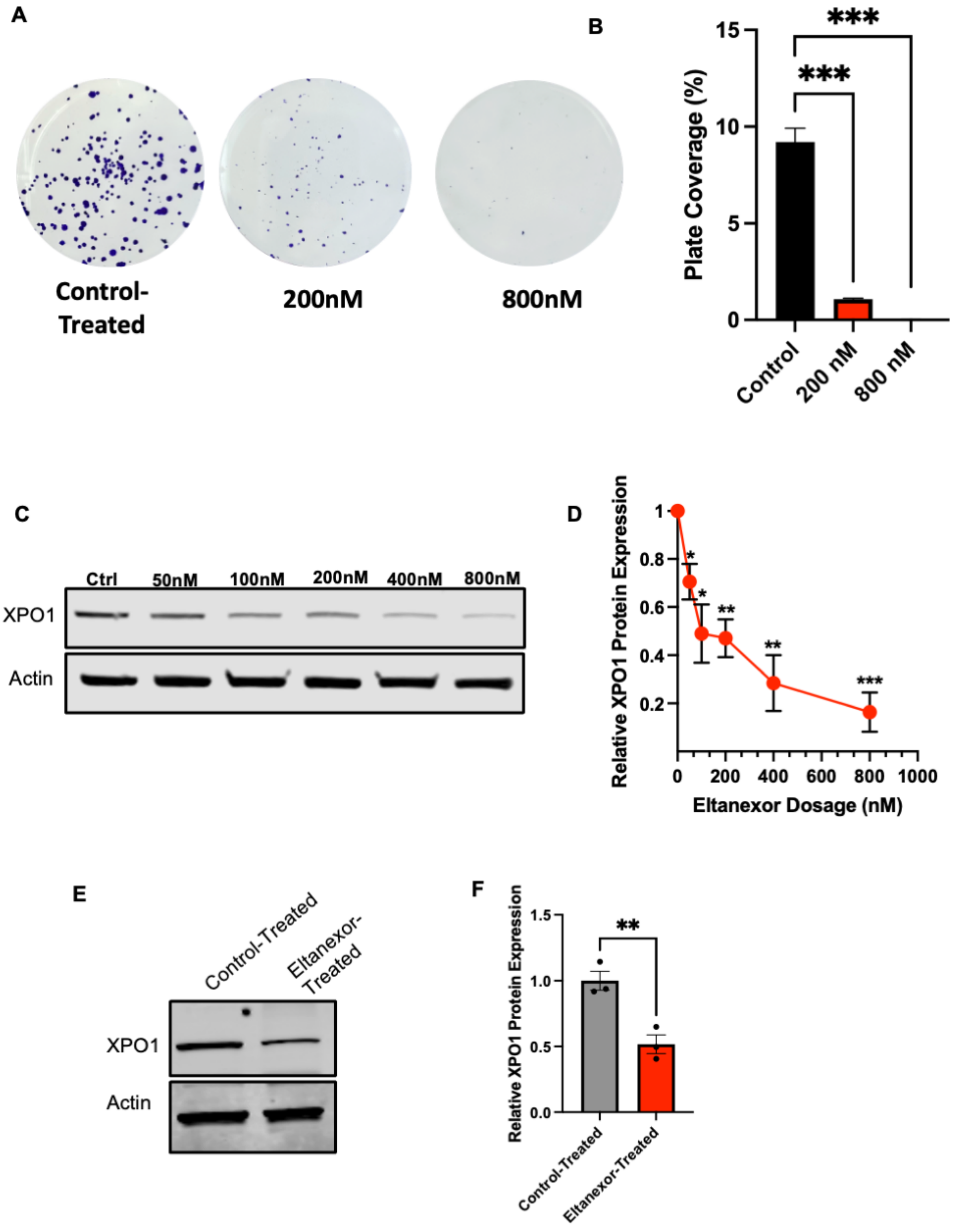

Supplementary Figure 2. Eltanexor treatment reduces XPO1 protein expression in multiple CRC cell lines and prevents colony formation. (A, B) HCT116 cells were plated with 500 cells/ well and treated with control, 200nM Eltanexor, or 800nM Eltanexor for 72 hours. The cells were cultured for an additional 8 days and stained with 0.1% crystal violet solution. The mean percent of the plate covered by colonies of 3 independent experiments ± SEM was determined. Student's t-test was used to statistically compare percent plate coverage in the control-treated vs 200nM Eltanexor-treated and the control-treated vs 800nM Eltanexor-treated groups. **(C, D)** XPO1

expression in HCT116 cells treated with varying doses of Eltanexor for 48 hours. The graph depicts normalized densitometry of immunoblot bands**.** The values graphed are the mean densitometry value of XPO1 bands normalized to actin and relative to control-treated cells of 3 independent experiments ± SEM. Student's t-test was used to statistically compare XPO1 protein expression in control-treated and Eltanexor-treated cells. **(D, E)** XPO1 expression in HCA7 cells treated with 200nM Eltanexor for 48 hours. The graph depicts normalized densitometry of immunoblot bands**.** The values graphed are the mean densitometry values of XPO1 bands normalized to actin and relative to control-treated cells of 3 independent experiments ± SEM. Student's t-test was used to statistically compare XPO1 protein expression in control-treated and Eltanexor-treated cells. *(*, $p \leq 0.05$; **, $p \leq 0.01$; ***, $p \leq 0.001$).*
