# Supplementary material for: The XPO1 Inhibitor Eltanexor Modulates the Wnt/β-Catenin Signaling Pathway to Reduce Colorectal Cancer Tumorigenesis
Source: Cancer Res Commun. 2025 Jul 15;5(7):1140–54. doi: 10.1158/2767-9764.CRC-25-0052 (PMC12260813; doi:10.1158/2767-9764.CRC-25-0052)
Supplement: Supplementary Figure 3 — Figure S3. Eltanexor treatment reduces HCT116 xenograft Growth. [file crc-25-0052_supplementary_figure_3_suppsf3.pdf]

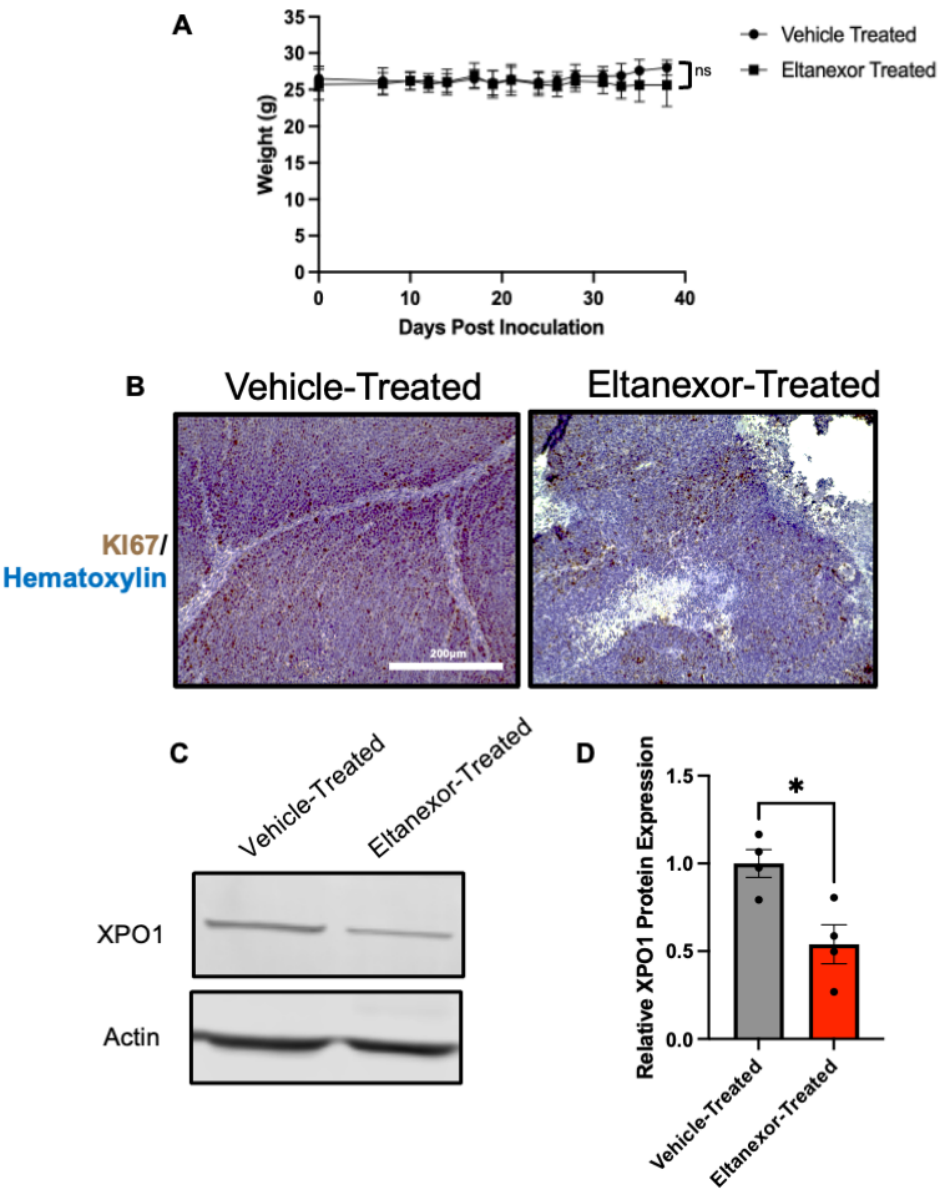

Supplementary Figure 3. Eltanexor treatment reduces HCT116 xenograft Growth. (A) Athymic mice injected with HCT116 cells and treated with either vehicle or Eltanexor weights over the course of the study. Each data point represents the mean mouse weight ± SEM. Student's t-test was used to statistically compare mouse weights in the vehicle-treated and Eltanexor-treated groups. **(B)** IHC detection of Ki67 in HCT116 xenograft tumors. Representative sections were stained for Ki67 (brown) and counterstained with hematoxylin (blue). The scale bars represent 200µm **(C, D)** Representative XPO1 protein expression from tumors derived from HCT116 xenograft mice treated with either vehicle or Eltanexor. The graph depicts normalized densitometry of immunoblot bands**.** The values graphed are the mean densitometry value of XPO1 bands normalized to actin and relative to control-treated cells of 3 independent experiments ± SEM. Student's t-test was used to statistically compare XPO1 protein expression in control-treated and Eltanexor-treated cells. *(*, p ≤ 0.05)*
