# Supplementary material for: The XPO1 Inhibitor Eltanexor Modulates the Wnt/β-Catenin Signaling Pathway to Reduce Colorectal Cancer Tumorigenesis
Source: Cancer Res Commun. 2025 Jul 15;5(7):1140–54. doi: 10.1158/2767-9764.CRC-25-0052 (PMC12260813; doi:10.1158/2767-9764.CRC-25-0052)
Supplement: Supplementary Figure 4 — Figure S4. Eltanexor does not changes β-catenin protein expression or intracellular localization. [file crc-25-0052_supplementary_figure_4_suppsf4.pdf]

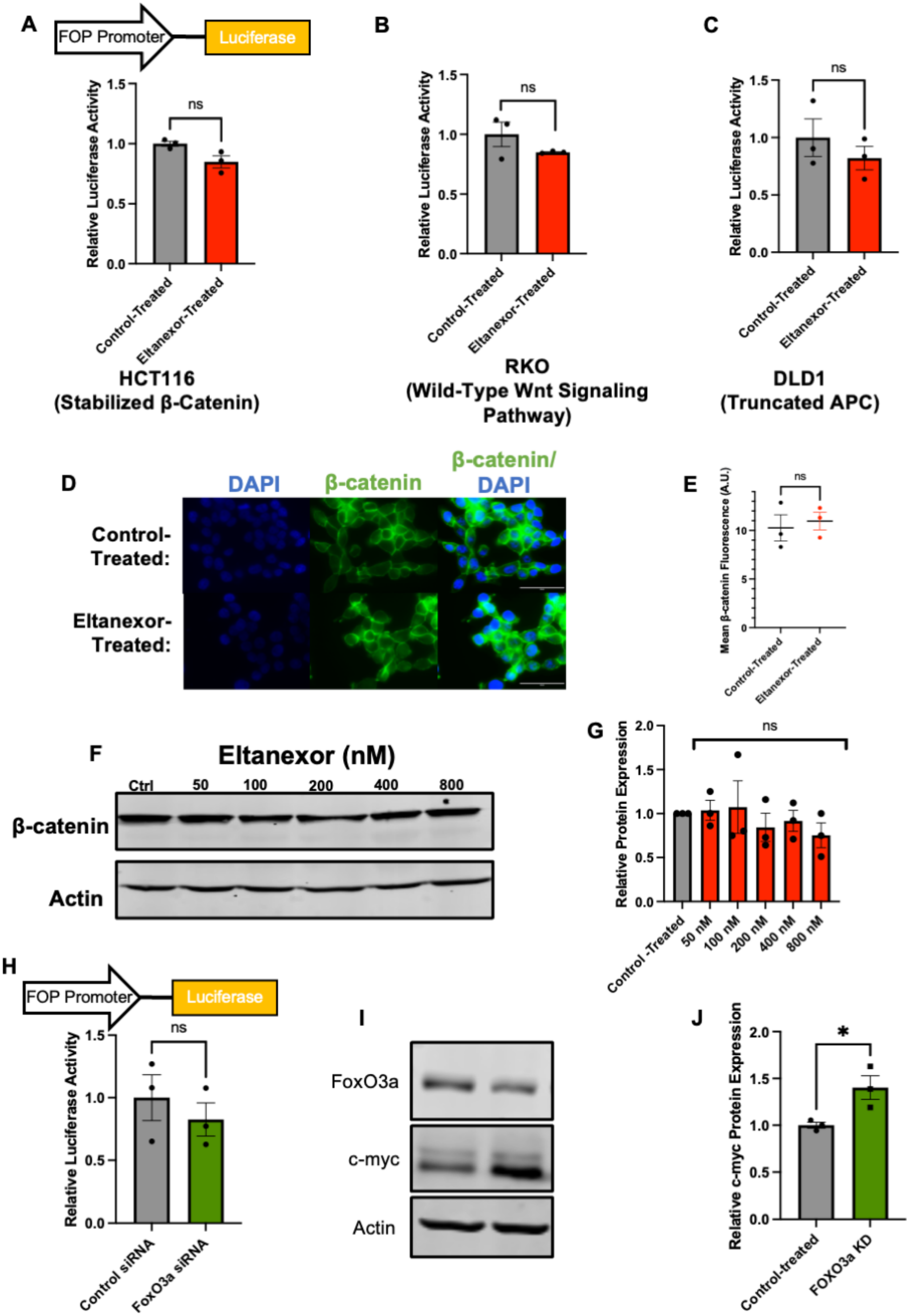

A
FOP Promoter
Luciferase
Relative Luciferase Activity
ns
Control-Treated
Eltanexor-Treated
HCT116
(Stabilized β-Catenin)
B
Relative Luciferase Activity
ns
Control-Treated
Eltanexor-Treated
RKO
(Wild-Type Wnt Signaling Pathway)
C
Relative Luciferase Activity
ns
Control-Treated
Eltanexor-Treated
DLD1
(Truncated APC)
D
DAPI
β-catenin
β-catenin/ DAPI
Control-Treated:
Eltanexor-Treated:
E
Mean β-catenin Fluorescence (A.U.)
ns
Control-Treated
Eltanexor-Treated
F
Eltanexor (nM)
Ctrl
50
100
200
400
800
β-catenin
Actin
G
Relative Protein Expression
ns
Control -Treated
50 nM
100 nM
200 nM
400 nM
800 nM
H
FOP Promoter
Luciferase
Relative Luciferase Activity
ns
Control siRNA
FoxO3a siRNA
I
FoxO3a
c-myc
Actin
J
Relative c-myc Protein Expression
*
Control-treated
FOXO3a KD

Supplementary Figure 4. Eltanexor does not changes β-catenin protein expression or intracellular localization. (A-C) HCT116, RKO, and DLD1 cells (all harboring different Wnt signaling phenotypes) were transfected with a FOPFlash reporter plasmid. Cells were subsequently treated with DMSO or Eltanexor. RKO and HCT116 cells were treated with 200nM Eltanexor, and DLD1 cells were treated with 400nM Eltanexor for 24 hours. Luciferase activity/ µg of protein was normalized to DMSO-treated luciferase expression for each respective cell line ± SEM. Student's t-test was used to statistically compare luciferase activity in the control-treated and Eltanexor-treated groups. **(D, E)** HCT116 cells were treated with 200nM Eltanexor or DMSO (control) for 48 hours. The cells were subject to immunofluorescent staining for β-catenin (green). The nucleus was stained with DAPI (Blue). The graph represents the comparison of β-catenin fluorescent signal between control-treated and 200nM Eltanexor-treated cells. The values graphed are the mean fluorescence values ± SEM. Student's t-test was used to statistically compare the control-treated group fluorescence to the Eltanexor-treated group's fluorescence. **(F, G)** HCT116 cells were treated with varying doses of Eltanexor or DMSO (control) for 48 hours. Subsequently, the cells were subjected to immunoblotting for changes in β-catenin protein expression. The graph depicts normalized densitometry of immunoblot bands**.** The values graphed are the mean densitometry value of β-catenin bands normalized to actin and relative to control-treated cells of 3 independent experiments ± SEM. Student's t-test was used to statistically compare β-catenin protein expression in control-treated and Eltanexor-treated cells. **(H)** In HCT116 cells, FoxO3a was knocked down by siRNA. Post-knockdown, the cells were transfected with a FOPFlash reporter plasmid. The relative luciferase activity/ µg of protein values were normalized to control siRNA-treated. Student's t-test was used to statistically compare luciferase activity in the control-treated and Eltanexor-treated groups. The graph depicts the mean of 3 independent experiments ± SEM. Student's t-test was used to statistically compare the control siRNA-treated and FoxO3a siRNA-treated groups. **(I, J)** Western blot depicting c-myc protein expression when FoxO3a is knocked down. The graph depicts normalized densitometry of immunoblot bands**.** The values graphed are the mean densitometry value of c-myc bands normalized to actin and relative to control-treated cells of 3 independent experiments ± SEM.
