# Supplementary material for: The XPO1 Inhibitor Eltanexor Modulates the Wnt/β-Catenin Signaling Pathway to Reduce Colorectal Cancer Tumorigenesis
Source: Cancer Res Commun. 2025 Jul 15;5(7):1140–54. doi: 10.1158/2767-9764.CRC-25-0052 (PMC12260813; doi:10.1158/2767-9764.CRC-25-0052)
Supplement: Supplementary Figure 5 — Figure S5. Eltanexor-treatment is well-tolerated in Apcmin/+ mice. [file crc-25-0052_supplementary_figure_5_suppsf5.pdf]

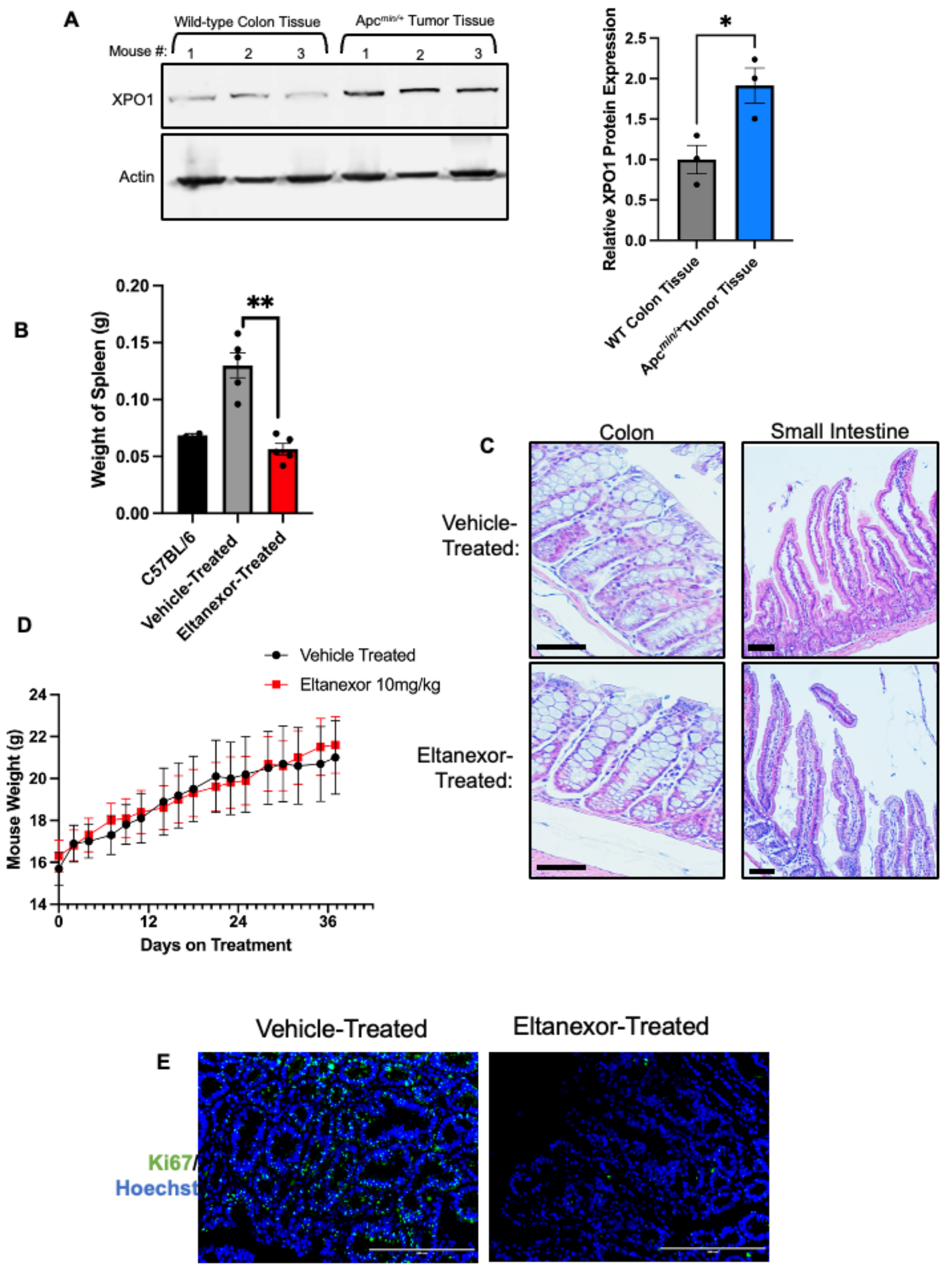

Supplementary Figure 5. Eltanexor-treatment is well-tolerated in Apc$^{min/+}$ mice. (A) Colon epithelial tissue from three wild-type C57BL/6 mice was collected, while colon tumors were removed from three Apc$^{min/+}$ mice. The tissues were then ran on a western blot to evaluate changes in XPO1 protein, with actin as the loading control. The graph shows the relative protein expression of XPO1 normalized to WT mice. Student's t-test was used to statistically compare XPO1 expression in the vehicle-treated and Eltanexor-treated groups. **(B)** Apc$^{min/+}$ mice were treated with either vehicle or 10 mg/kg of Eltanexor 3 days a week for 6 weeks. The weight of each mouse was recorded every Monday, Wednesday, and Friday. The values represent the

mean weight of the mice from each group on the given treatment date ± SEM. Student's t-test was used to statistically compare spleen weights the vehicle-treated and Eltanexor-treated groups. **(C)** Post-treatment, intestinal tissue was formalin-fixed paraffin-embedded. Representative Hematoxylin & Eosin (H&E)-stained mid-colon and proximal small intestine from mice either treated with vehicle or Eltanexor. The hematoxylin (purple) represents the nucleus, while the eosin (pink) represents the cytoplasm. Scale bars represent 50μm. **(D)** The mean spleen weight of C57BL/6 mice, vehicle-treated $Apc^{min/+}$ mice, and Eltanexor-treated $Apc^{min/+}$ mice ± SEM. Spleen weights of the C57BL/6 mice were age-matched to the treated mice. **(E)** Formalin-fixed paraffin-embedded tissue from vehicle-treated and Eltanexor-treated was immunofluorescent stained for Ki67 (green), and the nuclei were stained with Hoechst (blue). Scale bars represent 200 μm. *(*, p ≤ 0.05; **, p ≤ 0.01; ***, p ≤ 0.001)*
